# Molecular Detection of* Brucella* spp. from Milk of Seronegative Cows from Some Selected Area in Bangladesh

**DOI:** 10.1155/2018/9378976

**Published:** 2018-01-14

**Authors:** Md. Sadequl Islam, Md. Ariful Islam, Mst. Minara Khatun, Sukumar Saha, Md. Samiul Basir, Md- Mahmodul Hasan

**Affiliations:** ^1^Department of Microbiology and Hygiene, Bangladesh Agricultural University, Mymensingh 2202, Bangladesh; ^2^Hajee Mohammad Danesh Science and Technology University, Dinajpur 5200, Bangladesh

## Abstract

Brucellosis is endemic in Bangladesh both in humans and in animals. A number of reasons complicate the diagnosis, as bovine brucellosis can be diagnosed by various serological tests. But the tests have a limitation; when the organism remains intracellular, the disease goes into chronic stage and the antibody titres may decline. The present study was conducted for isolation and detection of* Brucella* spp. by polymerase chain reaction (PCR) from seronegative cows. A total of 360 dairy cows from three geographical regions were screened serologically by Rose Bengal Plate Test (RBPT) where 24 samples were serologically positive and the rest of the samples were serologically negative. Among the 24 seropositive individuals, 11 were culture positive and 6 were culture positive from serologically negative dairy cows. The overall seroprevalence of brucellosis in cattle was 6.6% and in disease condition a higher prevalence was recorded in abortion (28.07%) followed by infertility (13.33%). To confirm the* Brucella* spp. in seronegative dairy cattle, the isolates were extracted and PCR was conducted, which produced 905 bp amplicon size of 6* Brucella* spp. from milk sample. So, for the detection or eradication of brucellosis, a bacteriological test and a PCR technique should be performed with the serological test of milk.

## 1. Introduction

Brucellosis is one of the most important zoonotic diseases infecting humans and domesticated animals. It is endemic in many developing countries of Asia, Africa, and Latin America including Bangladesh. It is caused by a member of the Gram-negative bacteria that belongs to the genus* Brucella*. These are small, nonmotile, facultative anaerobic, intracellular, Gram-negative coccobacilli and show strong host preference [1, 2]. Five species of* Brucell*a are known to cause disease in domesticated animals such as* B. abortus *(cattle),* B. melitensis* (goats),* B. ovis* (sheep),* B. suis* (pigs), and* B. canis* (dogs). Human infections are caused by* B. melitensis*,* B. abortus*, and* B. suis* through direct contact with infected animals and drinking of unpasteurized or raw milk [3].

Brucellosis is transmitted through direct or indirect contact with infected animals “often via ingestion and also via venereal routes” [4]. The infection may occur less commonly via the conjunctiva and inhalation and in utero [5]. It can also be spread through fomites; the transmission of brucellosis by ticks, fleas, or mosquitoes from an infected herd to a noninfected herd has never been proven [6].

The World Health Organization (WHO) laboratory biosafety manual classifies* Brucella* in risk group III. Brucellosis is readily transmissible to humans, causing acute febrile illness and undulant fever, which may progress to a more chronic form and can also produce serious complications affecting the musculoskeletal, cardiovascular, and central nervous systems. Precautions should be taken to prevent human infection. Infection is often due to occupational exposure and is essentially acquired by the oral, respiratory, or conjunctival routes, but ingestion of dairy products constitutes the main risk to the general public where the disease is endemic. There is an occupational risk to veterinarians and farmers who handle infected animals and aborted fetuses or placentas. Brucellosis is one of the most easily acquired laboratory infections, and strict safety precautions should be observed when handling cultures and heavily infected samples, such as products of abortion. Specific recommendations have been made for the biosafety precautions to be observed with* Brucella*-infected materials [6].

Organisms remain alive for varying periods of time after elimination from animal body. This depends upon the environment and the keeping conditions. In suspensions,* Brucella *are killed in 20 minutes at 60°C. Direct sunlight for several hours is lethal to the organisms.* Brucella* will survive for a long time when exposed to cold temperatures.* Brucella abortus* will remain alive in the uterine discharge for up to 7 months after being stored in an ice chest. It remains viable for 30 days in ice cream stored at 32°F and in butter for 142 days kept at 46.5°F. It dies rather rapidly in milk at room temperature and survives longer at refrigerator temperature [7].

Brucellosis is endemic in Bangladesh [8, 9]. In Bangladesh, it was first reported in cattle in 1967 [10] and in human in 1983 [11]. Several studies were carried out in Bangladesh to record the seroprevalence of brucellosis in cattle and buffaloes [12–14].* Brucella abortus *has been identified by a real-time PCR assay directly from clinical specimens [15–17]. However, bacteriological identification of* Brucella* field isolate has not yet been performed. Milk is an important source of brucellosis in humans when they consume unpasteurized milk and undercooked milk. A number of circumstances complicate the diagnosis of Bovine brucellosis. The entrance of* Brucella* organism into the body can be diagnosed by a number of serological tests. But the tests have a limitation when the organism is harboured intracellularly and the disease goes into a chronic stage. In this situation, the antibody titres may decline or remain at the diagnostic threshold. Such type of animal may shed organisms in the milk, which is threatening for humans [18–20]. So, this study aimed to isolate* Brucella* spp. from seronegative individuals with a history of abortion, repeat breeding, stillbirth, and retention of placenta by using a standard cultural method to establish a base for epidemiological studies, management of outbreaks, and control and eradication programs of Bovine brucellosis in Bangladesh.

## 2. Materials and Methods

Blood and milk samples were collected from three geographical regions in Savar (23.8583°N 90.2667°E), Gazipur Sadar (24.0000°N 90.4250°E), and Mymensingh Sadar (24.7500°N 90.4167°E). A total of 360 milk and blood samples of each were collected from 22 dairy farms, and the history of the individuals was noted before collecting the samples.

About 5 ml of blood was collected from each of the individual cows after restraining and soaking the blood collection site at the jugular furrow with 70% alcohol. The collected blood samples with a syringe were kept undisturbed for about 4 hours at room temperature and then transferred to the refrigerator and kept overnight at 4°C. Later on, the sera were poured into an Eppendorf tube and were centrifuged at 2000 rpm for 10 minutes. After centrifugation, clear sera were obtained and then the sera were transferred to a sterilized labeled Eppendorf tube and stored at −20°C until use. Before beginning the Rose Bengal Plate Test (RBPT), the RBT antigen and the samples (sera) were kept at room temperature. The Rose Bengal Plate Test (RBPT) was performed to test the serum samples for the presence of* Brucella *spp. specific antibody according to the standard procedure of OIE (2008) [21].

During the collection of milk samples, the whole udder and teats of the cows were washed and dried. About 10 ml of milk was collected after discarding the first stream of milk from all quarters. Special care was taken to avoid contamination from the milker's hand. The collected milk samples in falcon tubes were kept in a refrigerator at 4°C overnight. Then, 1 ml of milk was put in each Eppendorf tube and centrifuged at 1500 rpm for 15 minutes at 4°C. The pellet and supernatants were collected in an Eppendorf tube for bacteriological analysis.

For the bacteriological study, 500 ml of* Brucella* selective agar media was prepared with the following composition:* Brucella* selective agar base: 22.5 mg, sterile sheep serum: 25 ml, and antibiotic (polymyxin B sulphate, bacitracin, nystatin, cycloheximide, nalidixic acid, and vancomycin): 10 ml. The serum was boiled at 55°C for 30 minutes, filtered with a 0.2 *μ*l sterile filter, and then mixed aseptically with* Brucella *agar base. The bacteria were isolated from milk samples using* Brucella* selective agar (HiMedia, Bombay, India). Processed milk samples were separately streaked onto* Brucella* selective agar media and inoculated culture media were placed in a CO_2_ incubator supplied with 5% CO_2_ at 37°C for 3–7 days. The plates were examined every 24 hours after 48 hours. With strict biosecurity measurement, the manipulation of clinical specimens for the isolation of* Brucella* spp. was performed in a biosafety class II A2 cabinet (Thermo Scientific, USA). Resultant colonies were subcultured and identified by cultural characteristics, Gram staining [22, 23] serum and carbon dioxide requirement for growth, hydrogen sulphide production, urease activity, and oxidase test. Representative colonies were stored at −80°C in 15% glycerol with trypto soya broth (TSB) for a long time for the preservation of the isolates.

DNA extractions were performed by Wizard® Genomic DNA Purification Kit (Promega Corporation). At first, a loop full of colonies was harvested and mixed well with 1 ml of phosphate buffer solution and centrifuged for 2 minutes at 13000 ×g. Then, the supernatant was discarded. Then, 600 *μ*l of a nuclei lysis solution was added and mixed by gentle pipetting. Then, it was incubated for 5 minutes at 80°C; then it was cooled to room temperature. Three-microliter RNase solutions were mixed and incubated at 37°C for 15–60 minutes and then cooled to room temperature. For protein precipitation, 200 *μ*l of a protein precipitation solution was added and vortexed properly. Then, it was kept on ice for 5 minutes and centrifuged at 13000 ×g. Then, for DNA precipitation and rehydration, the supernatant was transferred to a clean tube containing 600 *μ*l of isopropanol at room temperature and mixed properly. Then, it was centrifuged for 2 minutes at 13000 ×g and the supernatant was decanted. Then, 600 *μ*l of 70% ethanol at room temperature was added and mixed properly. It was centrifuged for 2 minutes at 13000 ×g and the ethanol was aspirated and the pellet air-dried for 10–15 minutes. The DNA pellet was rehydrated in 100 *μ*l of a rehydration solution for 1 hour at 65°C or overnight at 4°C.

A genus specific PCR assay was performed to identify* Brucella *spp. by amplifying a 905 bp fragment of the 16S* rRNA *gene encoding the heat shock protein [24]. Primers used in this PCR assay are F4(TCGAGCGCCCGCAAGGGG) and R2(AACCATAGTGTCTCCACTAA) [24]. The total volume of PCR mixture with PCR Master mixture (Thermo Scientific, USA) is 12.5 *μ*l, forward primer (20 pmol/*μ*l) is 1 *μ*l, reverse primer (20 pmol/*μ*l) is 1 *μ*l, template DNA is 5 *μ*l, and nuclease-free water is 5.5 *μ*l. The reaction was performed in a DNA thermal cycler at an initial denaturation temperature of 95°C for 5 minutes, followed by 35 cycles of denaturation at 95°C for 30 seconds, annealing at 54°C for 90 seconds, extension at 72°C for 90 seconds, and final extension at 72°C for 6 minutes. The amplified products were examined by electrophoresis in a 1.5% agarose gel and stained with ethidium bromide (0.5 mg/ml) in a dark place for 30 minutes. Then, the gel was destained in distilled water for 10 minutes and visualized under the UV transilluminator in the dark chamber of the image documentation system.

## 3. Results

**Figure 1:**
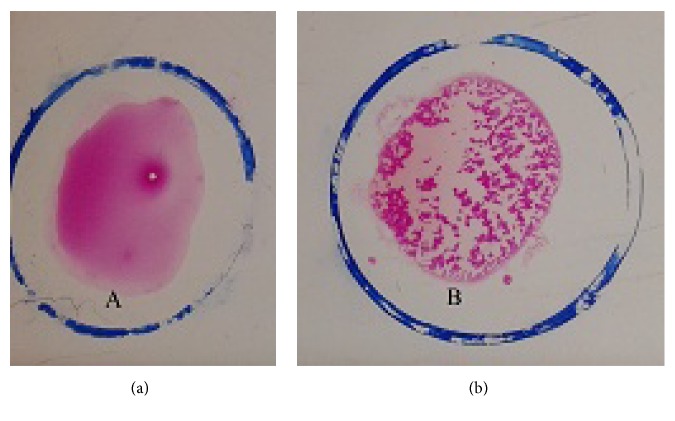
RBPT negative (a), RBPT positive (b).

From 360 tested samples, 24 (6.6%) were found to be serologically positive by RBPT and the rest of the cows were serologically negative by RBPT (Figure 1). Both samples were cultured in* Brucella* selective agar media and blood agar media. The results of RBPT and culture on media are shown in Table 1.

### 3.1. Overall Seroprevalence of Brucellosis in Cattle by RBPT

A total of 360 cattle sera were tested by RBPT where only 24 samples showed a positive reaction to RBPT (Figure 1). The overall seroprevalence of brucellosis in cattle was 6.6% (Table 2).

### 3.2. Seroprevalence of Brucellosis in Cattle according to Age

According to the age of the cattle, the older cattle showed higher prevalence than the younger one. Higher prevalence was seen more in cattle above 4 years old (8.18%) than the cattle 3 to 4 years of age (4.29%) (Table 3).

### 3.3. Seroprevalence of Brucellosis in Cattle Associated with Reproductive Disorders

On disease condition of the cattle, higher prevalence was recorded in case of abortion (28.07%) followed by infertility (13.33%) (Table 4).

### 3.4. Seroprevalence of Brucellosis in Cattle on the Basis of Pregnancy Status

Out of 360 cows, 87 were pregnant and 273 were nonpregnant and the prevalence of brucellosis was higher in pregnant cattle than in nonpregnant cattle (Table 5).

**Figure 2:**
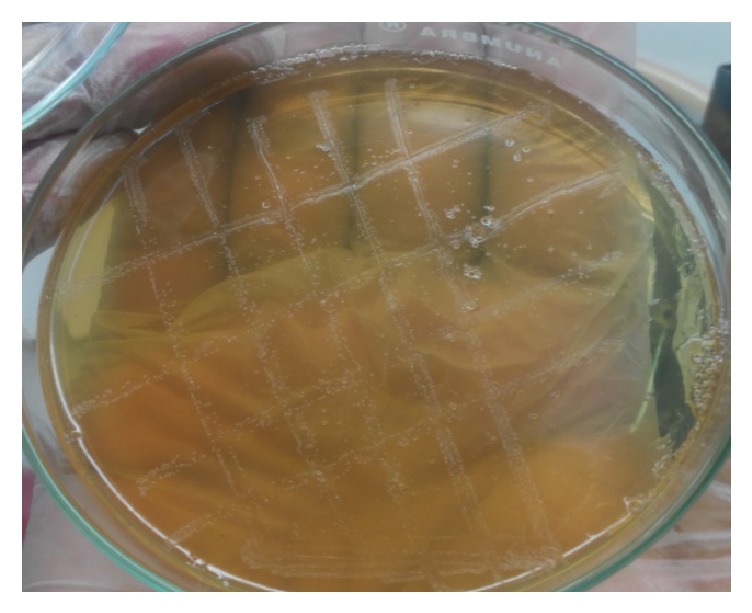
Small, translucent, dewdrop-like, round, and convex growth with smooth margins on* Brucella* selective agar media.

**Figure 3:**
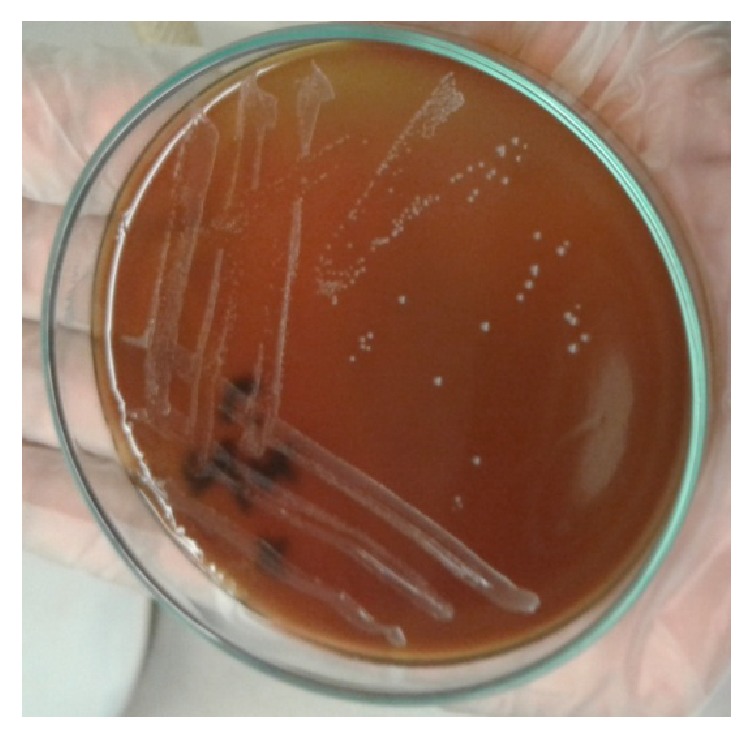
Whitish-grey, shiny, circular, convex, and nonhemolytic colonies of bacteria on blood agar media.

**Figure 4:**
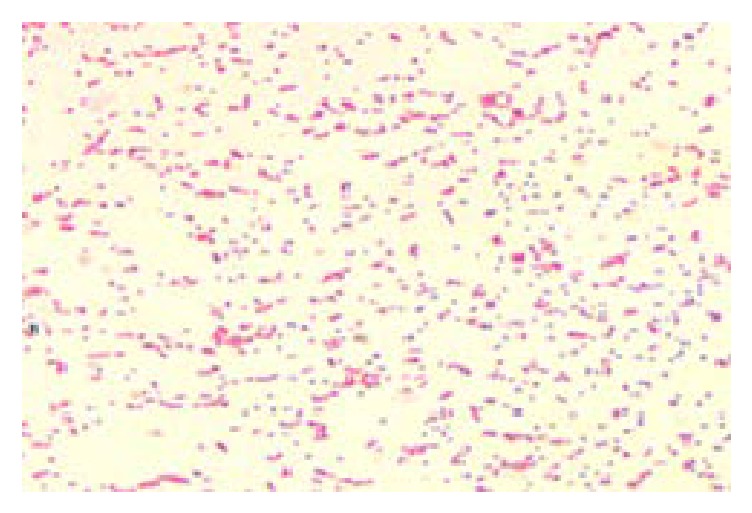
Gram-negative paired coccobacilli under a light microscope (400x).

Several colonies of* Brucella* spp. were observed on* Brucella* selective agar media after 3–5 days of incubation as small, translucent, dewdrop-like, round, and convex with smooth margins (Figure 2) and the cultural characteristics on blood agar whitish-grey, punctuate, shiny, nonhemolytic, and convex colonies were observed (Figure 3). The results of biochemical tests were recorded as follows: catalase test: positive, oxidase test: positive, MR test: negative, VP test: negative, indole test: negative reaction; and these are the characteristics of* Brucella *spp. In Gram's staining (Figure 4), bacterial isolates were seen as Gram-negative coccobacilli with single and pair arrangement.

**Figure 5:**
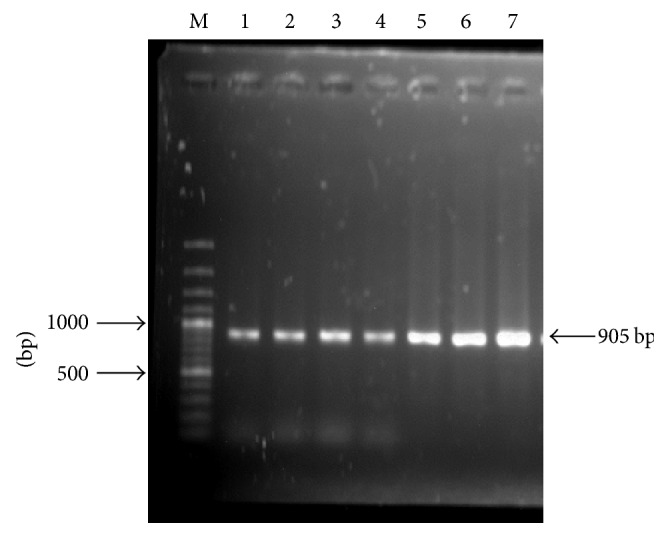
Agarose gel electrophoresis of PCR assay products. Lane M: 100 bp DNA ladder (Thermo Scientific); lanes 1–6: DNA of* Brucella* species from seronegative bovine milk (905 bp); lane 7: positive control.

Detection of* Brucella* spp. in milk samples from serologically negative cows by using genus specific primers (F4 and R2) targeting 16 SrRNA produced amplicons of 905 bp. Out of 342 serologically negative samples, there were 6 culture positive and amplified 905 bp PCR amplicons belonging to the genus* Brucella* (Figure 5).

## 4. Discussion

*Brucella* are always both intracellular and extracellular. The protective immunity is mainly cellularly mediated, but also humoral. The humoral immunity is useful for the indirect diagnosis by all the serological tests used: Rose Bengal Plate Test, complement fixation, and ELISA [25]. Diagnosis of brucellosis in bovine milk samples mainly depends on the milk ring test, which indirectly detects* Brucella* spp. in the host [26]. The* Brucella* organism in the body can be diagnosed by a number of serological tests, but the tests have a limitation when the organism is harboured intracellularly and the disease goes into the chronic stage. In this situation, the antibody titres may decline or remain at the diagnostic threshold and these animals may shed organisms in the milk which is threatening to humans [18–20]. In endemic areas, this organism is transmitted to people mostly through consumption of unpasteurized milk and milk products from sheep and goats [27–29]. During an investigation of bovine brucellosis in Iran, conducted by the Razi Institute over a twelve-month period, samples of serum and milk were collected simultaneously from 6,472 cows in eight infected herds for serological and bacteriological testing and 119* Brucella *spp. were isolated from 5686 seronegative cows, and the prevalence was 2.09% [30]. In the present, the prevalence of brucellosis in seronegative cows is 1.75% which is lower than in Zowghi et al. [30]. A study was conducted in Ethiopia in 2016 among 66 seronegative individuals; they cultured clinical samples, but all the samples were culture negative. In that study, no isolate was obtained from milk and fetal stomach content [31].

In cattle, it causes abortions, infertility, retention of the placenta, and stillbirth, resulting in huge economic losses in dairy industries [5, 32]. There are various eradication programs for controlling brucellosis which include vaccination, serological testing, and slaughtering [1, 2, 33, 34]. Regular serological surveillance is essential for undertaking control measures against brucellosis [35]. Each year, half a million cases of brucellosis are reported worldwide. Recently, many countries have eradicated brucellosis from their herds, and many other countries significantly reduced the prevalence of the infection among their livestock.

This study recorded 6.6% overall prevalence of* Brucella *spp. which is lower than the results of 12% [36], 8.1% [13], 7.76% [37], 15.33% [12], 14.14% [38], and 18.75% [39] and higher prevalence of brucellosis as compared to the reported 2.25% [40], 3.30% [41], 2% [42], 2.4% [43], 2.66% [44], and 2.72% [45] prevalence in cattle. The variation of prevalence of brucellosis might be due to difference of sample size, age, breed, pregnancy status of the animal, study area, hygienic condition, herd size, breeding techniques, reproductive diseases, and diagnostic tests [12, 46, 47].

In the study, the prevalence of brucellosis was found to be 4.29% in 3-4 years age group while it was 8.18% in the above 4 years age group. In contrast to the findings of the present study, 2.59% [41] prevalence of brucellosis was found in cows aged 2.5–4 years and 4.35% in cows over 4 years of age. Similarly, in 2005, prevalence was reported to be 2.3% and 4% [9] for lower than 4 and higher than 4 years age group, respectively. Susceptibility to disease increases with age; it seems to be more commonly associated with sexual maturity [5] and different studies reported that older animals are more susceptible than younger animals.

In the present study, the prevalence of brucellosis was higher (11.49%) than in nonpregnant cows (5.13%). Brucellosis causes abortion, retention of the placenta, repeat breeding, infertility, and prolonged intercalving period due to early embryonic deaths. This study recorded a prevalence of 28.07% brucellosis in cattle with a history of abortion and 13.33% brucellosis in cattle with a history of infertility, which agreed with the report of 2011 [14] which recorded the prevalence of brucellosis as 15% in cows that had a previous abortion, and the prevalence of brucellosis in repeat breeding cases was 45%. In 1975, the prevalence of brucellosis with a history of abortion was reported to be 14.2% [48], while in 2011 the prevalence of brucellosis with a history of retained placenta was stated to be 13.04% [14], whereas in the present study the prevalence was 5.71%.

However, in Bangladesh, the milk ring test is not practiced for the screening of* Brucella*. RBPT test is widely performed to detect the antibody of* Brucella* spp. in a herd. It is simple to perform, inexpensive, and suitable for screening individual animals. But RBPT may also produce false positive serological reactions with lipopolysaccharide (LPS) of* Yersinia enterocolitica 0: *9 and* Escherichia coli *0157: H_7_ or cross-reactive antigens from other bacteria such as* Salmonella *species and* Pasteurella *species [49–54]. For this reason to confirm an individual free from brucellosis, PCR technique is advisable.

## 5. Conclusion

In Bangladesh, in spite of the number of research works on seroprevalence of brucellosis in cattle and humans, there are no reports on bacteriological isolation and identification of* Brucella* spp. from serologically negative dairy cattle. In the present study,* Brucella* spp. were isolated from seronegative dairy cattle with a history of abortion, repeat breeding, retention of the placenta, and stillbirth. Hence, it is very important to isolate* Brucella* isolates to design an effective control measure for brucellosis in Bangladesh. So, it is advisable to detect or eradicate brucellosis; a bacteriological test and a PCR technique should be performed in addition to serological test of milk sample.

## Figures and Tables

**Table 1:** Results of RBPT and culture of seronegative dairy cows samples.

Name of the upazila	Total number of samples	RBPT +ve	Culture positive from RBPT positive milk samples	RBPT −ve	Culture positive from RBPT negative milk samples
Mymensingh Sadar	180	11	6	171	3
Savar	120	8	3	115	2
Gazipur Sadar	60	5	2	56	1

Total	360	24	11	342	6

+ve = positive, −ve = negative, and RBPT = Rose Bengal Plate Test.

**Table 2:** Overall seroprevalence of brucellosis in cattle.

Animal species	Number of sera tested	Number of positive reactors	Prevalence (%)
Cattle	360	24	6.6

**Table 3:** Seroprevalence of brucellosis in cattle according to age.

Animal species	Age of animal (years)	Number of sera tested	Number of positive reactors	Prevalence (%)
Cattle	3 to 4	140	6	4.29
	>4	220	18	8.18

**Table 4:** Seroprevalence of brucellosis in cattle associated with reproductive disorders.

Reproductive disorders	Number of sera tested	Number of positive reactors	Prevalence (%)
Abortion	57	16	28.07
Retention of placenta	35	2	5.71
Infertility	30	4	13.33
Metritis, repeat breeding, dystocia, and so forth	238	2	0.84

**Table 5:** Seroprevalence of brucellosis in cattle on the basis of pregnancy status.

Animal species	Pregnancy status	Number of sera tested	Number of positive reactors	Prevalence (%)
Cattle	Pregnant	87	10	11.49
	Nonpregnant	273	14	5.13

## References

[1] Baek B. K., Lim C. W., Rahman M. S., Kim C.-H., Oluoch A., Kakoma I. (2003). Brucella abortus infection in indigenous Korean dogs. *Canadian Journal of Veterinary Research*.

[2] Kakoma I., Oluoch A. O., Back B. K., Rahman M. S., Kiku M. (2003). More attention warranted on Brucella abortus in animals. *Journal of the American Veterinary Medical Association*.

[3] Corbel M. J., Thomas E. I. (2006). Use of phage for the identification of Brucella spp. *Research in Veterinary Science*.

[4] Quinn P. J., Carter M. E., Markey B., Carter G. R.

[5] Radostits O. M., Gaycc Blood D. C., Hinchcliff K. W. (1877). *Veterinary Medicine: A Textbook of The Diseases of Cattle, Sheep, Pigs, Goats and Horses*.

[6] OIE Terrestrial Animal Health Code Brucellosis. http://www.oie.int.

[7] Live I. (1954). *Public Health Reports*.

[8] Das T. M., Ershaduzzaman K. K., M Islam M., Hague M. M., Rahman I. C. B. M., Sailful I. (2008). Surveillance of Brucella melitensisand Brucella abortusfrom aborted bengal goats in Bangladesh. *Research Journal of Veterinary Science*.

[9] Amin K. M. R., Rahman M. b., Rahman M. S., Han J. C., Park J. H., Chae J. S. (2005). Prevalence of Brucella antibodies in sera of cows in Bangladesh. *Journal of VeterirnaryScience*.

[10] Mia A. S., Islam H. (1967). A preliminary study on the incidence of bovine infertility and economic loss caused by it. *Pakistan Veterinary Journal*.

[11] Rahman M. M., Choudhury M. R., Rahman A., Haque F. (1983). Seroprevalence of human and animal brucellosis in Bangladesh. *Indian Veterinary Journal*.

[12] Islam M. A., Akter L., Khatun M. M., Islam M. A. (2014). Seroprevalence of Brucellosis and its associated risk factors in bovine at greater Mymensingh district of Bangladesh. *Microbes and Health*.

[13] Ismail H. (2015). *Epidemiological investigation and molecular detection of Brucella spp. in cattle at Myemensingh [M.S. thesis]*.

[14] Rahman M. S., Faruk M. O., Her M. J. Y., Kim S. I., Kang S., Jung C. (2011). Sero prevalence of bovine brucellosis and its public health significance in selected sites of Bangladesh. *Veterinary Medicine*.

[15] Rahman A. K. M. A. (2015). *Epidemiology of brucellosis in humans and domestic ruminants in Bangladesh [Ph. D. Thesis]*.

[16] Rahman M. S., Sarker M. A. S., Rahman A. K. M. A. (2014). The prevalence of Brucella abortus DNA in seropositive bovine sera in Bangladesh. *African Journal of Microbiology Research*.

[17] Sarker M. A., Sarker R. R., Begum M. M. (2016). Seroprevalence and Molecular Diagnosis of *Brucella abortus* and *Brucella Melitensis* in Bangladesh. *Bangladesh Journal of Veterinary Medicine*.

[18] Brinley M. W. J., Macdiarmid A. (1960). The excretion of Brucella abortus in the milk of experimentally infected cattle. *Research of Veterinay Science*.

[19] Doyle T. M., Becket T. F. (1936). The isolation of Brucella abortus from the milk of cows with negative blood reaction to the agglutination test. *Journal of Comparative Pathology and Therapeutics*.

[20] Nicoletti P., Muraschi T. F. (1966). Bacteriologic evaluation of serologic test procedures for the diagnosis of brucellosis in problem cattle herds.. *American Journal of Veterinary Research*.

[21] Office International des Epizooties (2008). *Manual of Diagnostic Tests and Vaccines for Terrestrial Animals*.

[22] Barrow G. I., Feltham R. K. (1992). *Cowan and Steel's, Manual for the identification of medical bacteria*.

[23] Cheesbrough M. (2006). *District Laboratory Practice in Tropical Countries*.

[24] Romero C., Gamazo C., Pardo M., Lopez-Goni I. (1995). Specific detection of Brucella DNA by PCR. *Journal of Clinical Microbiology*.

[25] Pellerin J. L., Geral M. F., Lautie R. (1980). Le test immune-enzymatique ELISA, dans le diagnostic serologique de la brucellose humaine. *Revue Med Vet*.

[26] Godfroid J., Saegerman C., Wellemans V. (2002). How to substantiate eradication of bovine brucellosis when aspecific serological reactions occur in the course of brucellosis testing. *Veterinary Microbiology*.

[27] Banai M. (2002). Control of small ruminant brucellosis by use of Brucella melitensis Rev.1 vaccine: Laboratory aspects and field observations. *Veterinary Microbiology*.

[28] Godfroid J., Cloeckaert A., Liautard J.-P. (2005). From the discovery of the Malta fever's agent to the discovery of a marine mammal reservoir, brucellosis has continuously been a re-emerging zoonosis. *Veterinary Research*.

[29] Seleem M. N., Boyle S. M., Sriranganathan N. (2010). Brucellosis: A re-emerging zoonosis. *Veterinary Microbiology*.

[30] Zowghi E., Ebadi A., Mohseni B. (1990). Isolation of Brucella organisms from the milk of seronegative cows. *Revue scientifique et technique Office International Epizootic*.

[31] Geresu M., Ameni G., Wubete A., Kassa A. (2016). Isolation and Identification of Brucella Species from Dairy Cattle by Biochemical Tests: The First Report from Ethiopia. *World s Veterinary Journal*.

[32] Singh G., Sharma D. R., Sandhu K. S., Dhan N. K. Economic losses occurring due to bovine abortions in Punjab in.

[33] Matyas Z., Fujikura T. (1984). Brucellosis as a world problem.. *Developments in Biological Standardization*.

[34] World Health Organization (1986). Joint FAO/WHO Expert Committee on Brucellosis.

[35] Erdenebaatar J., Bayarsaikhan B., Yondondorj A. (2004). Epidemiological and serological survey of brucellosis in Mongolia by ELISA using sarcosine extracts. *Microbiology and Immunology*.

[36] Hasan M. M. (2016). *Seroprevalence and Molecular detection of Brucella spp. in cattle at the selected areas of Mymensingh District [M.S. thesis]*.

[37] Badal. (2014). Detection of *Brucella abortus* in raw milk by polymerase chain reaction assay.

[38] Deselegn T., Haimanot., Gangwar S. K. (2011). Seroprevalence study of bovine brucellosis in Assela government dairy farm of Oromia regional state, Ethiopia. *International Journal of Security and Network*.

[39] Abdel-Razik K. A., Ismail E. M., Youssef H. M., Hashad M. A. (2008). Diagnosis of brucellosis in dairy animals using nested polymerase chain reaction. *International Journal of Dairy Science*.

[40] Rahman M. S., Nuruzzaman M., Ahasan M. S. (2002). Prevalence of brucellosis in pigs: The First report in Bangladesh. *Bangladesh Journal of Veterinary Medicine*.

[41] Nahkur E., Ernits E., Jalakas M., Järv E. (2011). Sero prevalence of bovine brucellosis and its public health significance in selected sites of Bangladesh. *Veterinary Medicine*.

[42] Kadir (2010). Seroprevalence of Brucellosis in cattle in some of the selected areas of Naogaon District.

[43] Nahar A., Ahmed M. (2009). Seroprevalence study of brucellosis in cattle and contact human in Mymensingh district. *Bangladesh Journal of Veterinary Medicine*.

[44] Nur-Alam (2008). Seroprevalence of specific Brucella infection of cattle in BAU veterinary clinic and its surrounding areas.

[45] Rahman M. S., Han J.-C., Park J., Lee J.-H., Eo S.-K., Chae J.-S. (2006). Prevalence of brucellosis and its association with reproductive problems in cows in Bangladesh. *Veterinary Record*.

[46] Kebede T., Ejeta G., Ameni G. (2008). Seroprevalence of bovine brucellosis in smallholder farms in central Ethiopia (Wuchale-Jida district). * Revue de Médecine Vétérinaire*.

[47] Pandey H. S., Desai K. N. (1973). Incidence of abortion in cross-bred cows in heavy rainfall areas. *Indian Veterinary Journal*.

[48] Ibrahim A. E., Habiballa N. (1975). A survey of brucellosis in Messeriya cows of Sudan. *Tropical Animal Health and Production*.

[49] Food and Agricultural Organization (1986). FAO-WHO expert committee on brucellosis.

[50] Nielsen K. (1990). The Serological Response of Cattle Immunized with *Yersinia enterocolitica* O:9 or O:16 to *Yersinia* and *Brucella abortus* Antigens in Enzyme Immunoassays. *Veterinary Immunology and Immunopathology*.

[51] Nielsen K. (2002). Diagnosis of brucellosis by serology. *Veterinary Microbiology*.

[52] Nielsen K. H., Kelly L., Gall D., Nicoletti P., Kelly W. (1995). Improved competitive enzyme immunoassay for the diagnosis of bovine brucellosis. *Veterinary Immunology and Immunopathology*.

[53] Nielsen K., Smith P., Widdison J. (2004). Serological relationship between cattle exposed to *Brucella abortus, Yersinia enterocolitica* O:9 and Escherichia coli O157:H7. *Veterinary Microbiology*.

[54] Nielsen K., Smith P., Yu W. (2006). Serological discrimination by indirect enzyme immunoassay between the antibody response to Brucella sp. and Yersinia enterocolitica O:9 in cattle and pigs. *Veterinary Immunology and Immunopathology*.

